# AOW-YOLO: An efficient and lightweight model for smoking behavior detection on construction sites

**DOI:** 10.1371/journal.pone.0347052

**Published:** 2026-05-06

**Authors:** Ruishi Liang, Shuo Li, Shuaibing Li

**Affiliations:** School of Computer, University of Electronic Science and Technology of China, Zhongshan Institute, Zhongshan, China; Jiangsu Open University, CHINA

## Abstract

We propose AOW-YOLO, a novel model designed to address the challenge of detecting smoking behavior in complex construction site environments, which is particularly difficult due to the typically small size of objects and cluttered backgrounds. AOW-YOLO is built upon YOLO11n through the following steps. First, we develop a novel loss function, Adaptive Occlusion and Weighting IoU (AOWIoU), which dynamically optimizes sample quality gradient allocation using multiple mechanisms, thereby overcoming the reduced sensitivity of CIoU to spatial scale errors. Second, we introduce the Spatial Grouped-Pointwise (SGP) convolution module. By integrating a channel-adaptive grouping mechanism, it minimizes information loss during downsampling, alleviating issues related to low resolution and feature degradation. Finally, our SGP module replaces the original backbone in LCNet. Through pointwise convolution, this method enables cross-group information fusion and expands channel dimensions, effectively solving feature integration problems caused by excessive channel segmentation. Experimental results show that on the smoking detection dataset, the AOW-YOLO model surpasses existing lightweight models (e.g., PP-LCNet) in both mAP50 and mAP50:95 metrics, while achieving roughly 31.6% faster inference speed than YOLO11n. This approach provides new insights for designing lightweight detection models and has broad potential applications in construction site safety management.

## Introduction

In complex environments like building sites, unextinguished cigarette butts significantly increase fire risk, leading to secondary accidents that threaten safety and cause disruptions. Therefore, most construction sites enforce strict no-smoking policies. However, violations persist, resulting in substantial economic losses. Developing an effective system to detect and alert violations has become urgent to improve safety standards and risk management at construction sites.

Traditional detection methods have certain advantages but also face notable limitations. For example, manual patrols and passive video review not only experience low detection efficiency and high missed incident rates but also struggle to provide real-time reporting and emergency response. Vidhatri et al. [1] proposed a smoke-sensor-based fire prevention and control system that can transmit fire information in real time and accurately locate the ignition source, demonstrating high timeliness and accuracy in fire response. However, this approach only reacts after a fire begins, making preemptive prevention difficult. Additionally, deployment costs are substantial, potentially reaching millions of dollars per factory. Imtiaz et al. [2] conducted a systematic evaluation of the latest wearable smoking behavior sensors. While these sensors show high accuracy under laboratory conditions, their practicality and cost-effectiveness are limited in complex environments such as construction sites.

Object-based detection methods balance real-time performance with cost-efficiency, showing high potential and scalability for smoking detection tasks. Compared to traditional patrol inspections or single-sensor solutions, these strategies allow direct localization and identification of smoking-related objects within video streams, enabling comprehensive online analysis. However, when used in actual construction sites, they face several challenges: extreme lighting changes (such as overexposure, underexposure, reflections, or backlighting); various occlusions and dense interactions between targets; small object sizes and the absence of detailed features in long-distance imaging; pseudo-similarity interference within complex backgrounds; and hardware limitations on edge devices, including computational power, energy use, and latency. For example, Zhang et al.’s DAHD-YOLO [3] aims to work well across different environments, showing increased resilience under harsh lighting conditions (like strong backlighting, localized highlights, or low light) while maintaining fairly stable detection in visually tough situations. Still, its high overall computational complexity and resource needs, due to complex network design and processing paths, restrict its use in lightweight and low-power settings. Conversely, Jia et al.’s GeoIoU-SEA-YOLO [4] performs well in detecting small objects, handling occlusions, and modeling multiple object interactions, improving recall and robustness in challenging environments. Yet, when run on edge devices, limitations in computational capacity and bandwidth cause delays and slower inference speeds, preventing it from meeting strict real-time demands and reducing its usefulness for instant alerts in large-scale, affordable applications.

Notably, the complexity of both the scene and the data amplifies these challenges: in open environments like construction sites, cameras are typically placed at ground level to monitor workers performing elevated tasks. Because of long shooting distances, many obstructions, and mainly upward-facing angles, the proportion of effective positive samples is often low. This factor directly weakens the classification ability of detection models. During data annotation and sample construction, cigarettes rarely appear as standalone objects but are part of composite entities—such as being held between lips or fingers. Annotation boxes therefore inevitably include non-critical areas like fingers or lips. This accompanying information can be mistakenly learned by models as “cigarette” features during training, disrupting the model’s representation of the cigarette itself and reducing its generalization performance in real-world scenarios. During actual deployment, detection models are also vulnerable to interference from pseudo-similar targets. Common examples include slender, uniformly textured objects like fingers, pens, railings, or screwdrivers, which are often misclassified as cigarettes. Such confusion leads to false positives, false negatives, and overall accuracy decline. Dynamic factors at construction sites further worsen this challenge. Frequent personnel movement, cluttered backgrounds, and interference from dust and smoke all increase false positive rates while undermining detection stability and reliability. At the same time, cigarettes—as small objects—occupy a very low number of pixels in images. Under conditions of long-range capture and scale variation, they often lose fine details. Existing detectors find it hard to model these local features properly, greatly raising the risk of false negatives.

Based on the issues mentioned above, this paper introduces the enhanced model AOW-YOLO built on YOLO11n. Its main contributions are as follows:

(1) We propose a new regression loss function, AOWIoU (Adaptive Occlusion and Weighting IoU), to replace the original CIoU loss function in YOLO11n. This function includes a dynamic threshold mechanism and an occlusion-aware module within the WIoUv3 [5] framework, effectively suppressing the dominant influence of both high-quality and low-quality extreme samples during gradient updates and reducing the noise amplification effect. At the same time, this loss function applies moderate weight enhancement to ambiguous and occluded samples. This helps to reduce classification confusion and false detections caused by local feature interference, significantly boosting the model’s detection robustness in densely occluded scenarios.
(2) We propose a novel downsampling convolution module called Spatial Grouped-Pointwise (SGP). This module first uses Space-to-Depth (SPD) to perform lossless spatial-to-channel rearrangement of features, transferring fine-grained spatial information to the channel dimension; then, it introduces channel-adaptive grouped convolutions to expand the channel dimension and model local dependencies; finally, pointwise convolutions enable cross-group information fusion and further expand the channel dimension. As a result, SGP effectively reduces feature isolation and detail loss while replacing traditional stride = 2 convolutions.
(3) We propose a new backbone network called Lightweight CPU-Friendly Fast Net (LCFNet). To achieve lightweight and efficient inference on CPUs, we take inspiration from PP-LCNet [6], a backbone network based on the MKLDNN [7] acceleration strategy. Cui et al. developed the DepthSepConv module for backbone network reconstruction. Building on this, we further prune redundant convolutional layers to lower computational complexity. At the same time, we replace all stride = 2 convolutions with SGP module to reduce the loss of fine-grained information caused by downsampling. These improvements enhance feature learning while keeping a compact number of parameters, thereby increasing CPU-based inference efficiency.

Through the work described above, the model proposed in this paper achieves significant reductions in parameters and FLOPs while enhancing detection performance for small objects, occlusion, and dense scenes. It allows for the combined optimization of high accuracy and real-time capability while maintaining low deployment costs.

## Related works

### Two-stage versus one-stage detectors: Differences in paradigms and application limits

With advancements in computer vision and deep learning, object detection models have developed into two main paradigms: two-stage and one-stage approaches. Two-stage detection models(R-CNN [8], Fast R-CNN [9], Faster R-CNN [10], Mask R-CNN [11]) initially generate candidate regions, then perform detailed classification and bounding box regression. However, their high computational costs and slow inference speeds limit their use in edge devices and real-time applications. Conversely, one-stage detection models, especially the YOLO series [12] algorithms, have gained notable attention because of their low computational requirements, rapid inference speeds, and strong real-time capabilities. These benefits have shown high practical value in detecting smoking behaviors in construction site scenarios. Ji et al. proposed YOLO – TLA [13], which improves YOLOv5 for small – object detection by adding an additional tiny detection layer and introducing C3CrossConv along with a Global Attention Mechanism (GAM) to enhance feature extraction and detection performance.

### Lightweight backbones and heavy structural parametrization: Balancing real-time performance with representation power

To meet the inference demands of low-computing-power devices, researchers continue exploring lightweight solutions by refining backbone networks(e.g., GhostNet [14], ShuffleNet [15], FasterNet [16], PP-LCNet). Many architectures reduce FLOPs through techniques like separable convolutions, pointwise convolutions, group convolutions, and feature reuse, while improving parallelism and cache efficiency on CPU and GPU heterogeneous hardware. Combined with structural reparameterization during training and inference, new branches can be added during training to boost representational capacity, then folded into equivalent lightweight computation graphs during inference to decrease detection latency. It is important to note that lightweighting often causes side effects such as insufficient learning of channel-mixed features and reduced long-range context modeling capabilities. This is especially true in scenarios involving small objects, crowded environments, and strong occlusions, where representation deficiencies are more likely to occur. Achieving a controllable balance among bandwidth, computational power limits, and feature expressiveness remains a key challenge for edge deployment.

### Downsampling strategies and fine-grained information preservation: From *stride* = 2 to SPD reshaping

Hierarchical feature representations rely on stepwise downsampling to expand receptive fields and reduce computational complexity. However, traditional convolutions or pooling with *stride* = 2 cause a loss of fine-grained spatial features, impairing the distinguishability of small, elongated objects such as cigarettes. To address this issue, Sunkara et al. proposed SPDConv [17], a Space-to-Depth (SPD) approach. By reshaping neighboring pixels into the channel dimension, it preserves more local details without adding extra information loss. This method performs well under low-resolution and small-object conditions. However, the direct “spatial → channel” mapping is limited by cross-channel interactions and adaptive modeling capabilities: if subsequent convolutions or attention mechanisms do not achieve adequate cross-channel fusion, the high-dimensional channel features after reshape may remain fragmented, which hampers the efficiency of downstream multi-scale fusion.

### Bounding box regression loss: From geometric constraints to quality perception

Bounding box regression directly affects the final localization performance. The classic IoU loss produces zero gradients when predicted boxes do not intersect with ground truth boxes (IoU = 0), resulting in a lack of effective guiding gradients during early training or when initialization errors are large, which leads to slow convergence. To address this, subsequent work introduced a “geometric constraint enhancement” approach. GIoU [18] adds a minimum bounding rectangle penalty on top of IoU, allowing non-zero gradients even when boxes do not intersect. Building on this, DIoU [19] further incorporates a center point distance term to strengthen the geometric relationship between ground truth and predicted boxes and speed up convergence. Later, CIoU adds aspect ratio consistency into the model, making the regression target more closely match the shape of the ground truth bounding box; to unify multiple geometric factors, SIoU [20] models angle, distance, and shape relationships to improve geometric optimization. Conversely, EIoU [21] simplifies the regression target by directly minimizing width and height differences, boosting optimization efficiency. Unlike the “geometric term design” approach, the WIoU series shifts focus to “sample-quality-based gradient allocation.” By using quality-aware weights and dynamic re-weighting, it reduces the impact of extremely high- or low-quality samples on the optimization process, promoting effective learning from medium-quality samples and improving overall robustness. However, in complex scenarios with occlusion, blurring, and dense overlaps, IoU only measures overlap between predicted and ground-truth boxes and cannot fully capture the learning value of samples. This creates a gap between geometric metrics and learning needs, causing appearance-degraded samples to be misclassified as “low-quality” and overly suppressed. As a result, it hinders thorough learning of critical challenging samples.

### Adaptive meta-learning and feature regularization: Task-aware and channel-wise enhancement

Wang et al. proposed FRCAE [22], a feature regularization meta-learning framework with channel-wise attention expansion that increases feature dimensionality via self-attention, and further regularizes learning by minimizing the KL divergence between the query and support sets to encourage consistent and transferable representations. Wan et al. introduced TSML [23], a task-adaptive selection meta-learning algorithm that assesses task difficulty and potential future returns; it feeds task information into a multi-armed bandit to select the most beneficial task for inner-loop training and adjusts the outer-loop update strategy to improve generalization and convergence.

### YOLO11

**Fig 1:**
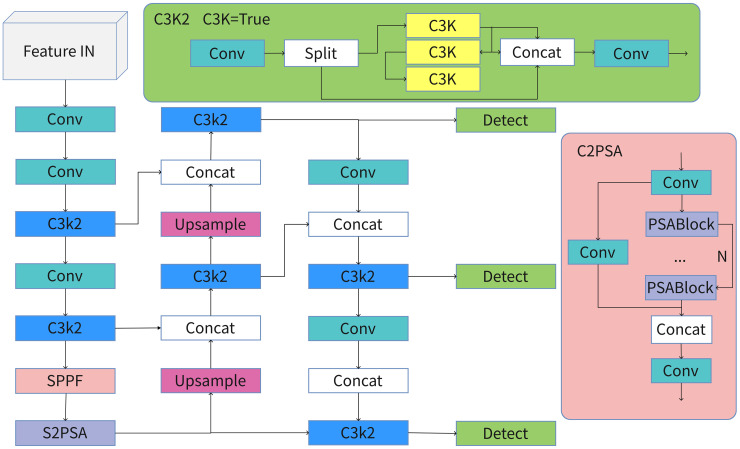
The architecture of YOLO11.

In the field of computer vision, the YOLO series has achieved notable success. Building on this foundation, researchers continue to improve the series through architectural advancements and module enhancements. Ultralytics released YOLO11 on September 27, 2024. Compared to earlier YOLO variants, YOLO11 strikes a balance between accuracy and speed across various tasks, including object detection, instance segmentation, pose estimation, image classification, and Oriented Bounding Box (OBB) detection. It incorporates systematic optimizations at the backbone, neck, and detection head levels, with its neural network architecture shown in Fig 1.

Regarding the backbone network, YOLO11 uses lightweight units to improve feature representation from shallow to intermediate layers while reducing redundant computations. This provides stable multi-scale inputs for subsequent FPN/PAN [24,25] (Feature Pyramid Network/Path Aggregation Network) fusion and detection heads. Throughout multiple stages, YOLO11 employs C3K2 as the main repeating unit (and partial neck) to replace or enhance existing C2f/C3 structures. This results in better local-global information coupling and feature reuse efficiency while maintaining overall computational complexity. C3K2 inherits the “branching-and-aggregation” design of CSP (Cross-Stage Partial), while improving gradient propagation and enhancing contextual modeling through optimized convolutional-and-pooling branch combinations. As a result, it is widely used as the backbone repeatable unit in several improved models.

In its neck architecture, YOLO11 uses a combined FPN/PAN path that integrates bottom-up and top-down approaches, emphasizing cross-layer and cross-scale information fusion. In serial implementations, it is often co-optimized with the backbone. Through efficient feature aggregation operators, attention mechanisms, and reparameterization strategies, the model improves robustness to small objects, dense clusters, and complex scenarios (e.g., occlusions and scale variations) while maintaining detection rates. It also offers unified feature support for multi-task learning (detection, segmentation, pose estimation, and OBB).

Regarding the detection head, YOLO11 uses an anchor-free and decoupled head approach: the classification and regression branches work independently, with location regression focused around center points and scales. This design lowers dependence on anchor priors and complex matching methods, usually speeding up convergence, enhancing generalization, and making deployment easier. This approach became popular starting with YOLOv8 and is further improved in YOLO11; related enhancements are generally confirmed through metrics like mAP, FLOPs, parameters, and FPS.

## Method

**Fig 2:**
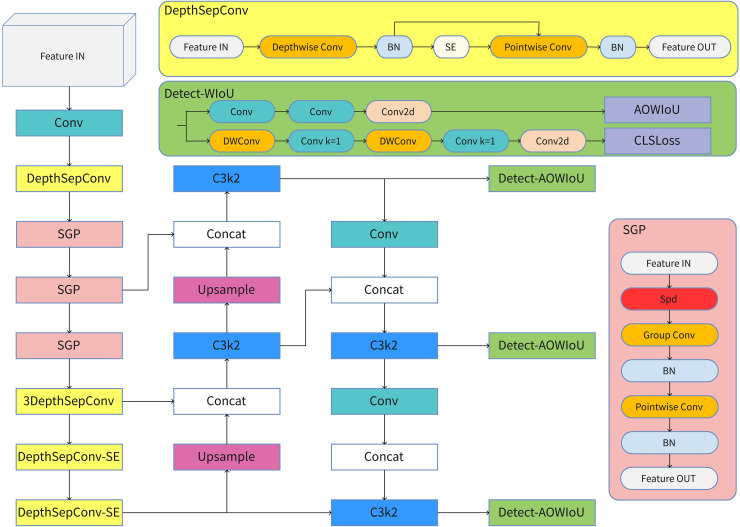
The architecture of AOW-YOLO.

To address challenges such as small objects, limited feature representation, and complex environments in construction site smoking scenarios, this paper introduces an improved model—AOW-YOLO. The model enhances both network architecture and loss functions. First, for regression optimization, we designed a novel AOWIoU loss function to reduce gradient bias toward low-quality and high-quality extreme samples. We also added an occlusion-aware mechanism to improve detection robustness in complex scenarios. Second, for downsampling, we proposed a new convolutional module, SGP, which effectively minimizes feature loss and isolation effects while reducing computational complexity. Finally, for the backbone network, we designed a lightweight backbone, LCFNet, by removing redundant convolutional layers from PP-LCNet and using the SGP module for downsampling. The overall neural network architecture is shown in Fig 2.

### AOWIoU

**Fig 3:**
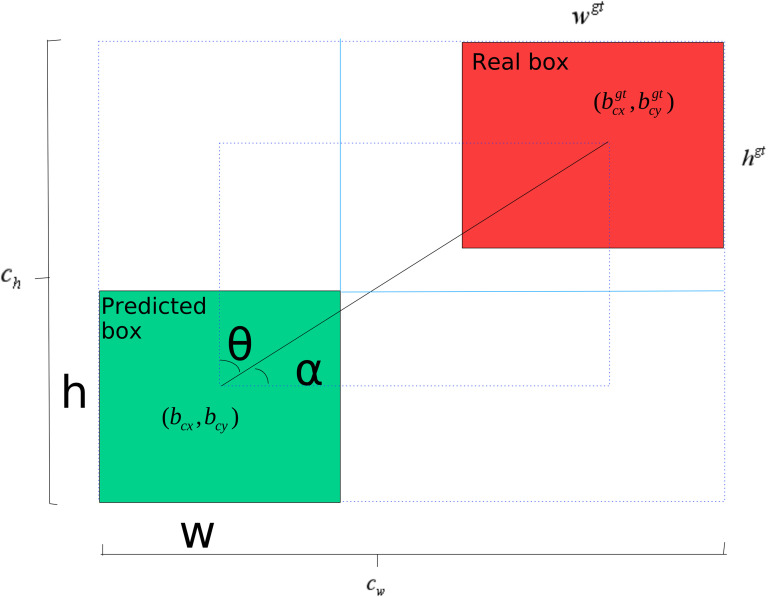
Illustration of the Loss Function. Intersection over Union (IoU) represents the ratio of the intersection between the predicted bounding box and the ground truth bounding box. Parameters involved in formula 1: $\rho (b,b^{gt})$ denotes the Euclidean distance between the centroid of the ground truth box and the predicted box; *h* and *w* represent the height and width of the predicted box; *h*^*gt*^ and *w*^*gt*^ denote the height and width of the ground truth box; *c*_*h*_ and *c*_*w*_ denote the height and width of the minimum bounding box formed by the Prediction Box and Real Box.

YOLO11n employs CIoU (Complete Intersection over Union) as the loss function for bounding box regression. Compared to traditional IoU, it introduces three constraints—overlap, center distance, and aspect ratio consistency—during target box optimization. This approach provides directional geometric gradients during non-overlap or low-overlap phases, speeding up convergence and improving early training stability. Real box and predicted box parameters are presented in Fig 3. Its definition is shown in Equation 1:


$${ℒ}_{CIoU}=1-IoU+\frac{\rho^{2}\;\left(b,\;b^{gt}\right)}{(c_{w}{)}^{2}+(c_{h}{)}^{2}}+\frac{4}{\pi^{2}}\;\left(\tan^{-1}\;\frac{w^{gt}}{h^{gt}}-\tan^{-1}\;\frac{w}{h}\right)$$ (1)

However, in complex scenarios like construction sites where computational resources are limited, and small objects, severe occlusions, and high viewing angles reduce sample feature quality, this loss function shows some limitations. First, CIoU treats all training samples equally without a mechanism to dynamically adjust weights based on sample difficulty. This fixed-weight approach can cause the model to overfit and ignore challenging samples with blurred boundaries or large deviations, which weakens overall detection robustness. Second, its aspect ratio penalty design has structural flaws: when predicted and ground truth boxes have the same aspect ratio but differ greatly in width and height, CIoU’s penalty does not accurately reflect the true geometric difference between them. This reduces the loss function’s sensitivity to spatial scale errors. Additionally, CIoU involves complex mathematical operations such as inverse tangent (arctangent), which increase computational overhead and limit its performance on resource-constrained edge devices for real-time tasks.

Traditional IoU-based regression losses implicitly treat training samples as independent and equally informative. However, in real-world detection tasks, such as monitoring smoking behavior on construction sites, targets are often partially occluded by surrounding structures (e.g., arms, tools, scaffold components), blurred by motion, or observed under extreme viewing angles. In these cases, a predicted box may exhibit similar geometric consistency with multiple nearby objects, creating ambiguous correspondence between the prediction and the intended target. This overlap competition introduces confusion-induced gradient noise: localization updates may be driven by cues from an unintended reference object, reducing localization stability and increasing jitter in cluttered scenes. Although WIoUv3 employs a non-monotonic dynamic weighting mechanism to emphasize medium-quality samples, it still does not explicitly distinguish between clean, learnable medium-overlap samples and visually ambiguous medium-overlap samples caused by occlusion, blur, or perspective distortion. Consequently, samples with comparable overlap quality can provide vastly different supervision reliability: clear instances offer consistent gradients that help refine boundaries, whereas ambiguous instances contain weak or conflicting visual evidence and are more likely to mislead optimization. This motivates an ambiguity-aware regression strategy that explicitly suppresses uncertain supervision and prioritizes samples with interpretable geometric cues, thereby improving localization robustness in dense and occluded construction-site scenarios.

To address the limitations of conventional CIoU, our AOWIoU (Adaptive Occlusion and Weighting IoU) builds on the dynamic weighting concept of WIoUv3 by introducing an occlusion-aware factor and a strategy to improve medium-quality samples: Low-quality samples are suppressed via thresholds, while fuzzy and partially occluded samples receive moderate weighting. At the same time, the gradient contribution of high-quality samples is limited to balance optimization across different quality levels and stabilize the regression surface. The interaction with the Consistent Dual Assignment (CDA) training process further ensures consistent positive/negative sample allocation across classification and regression branches, reducing label noise interference in the optimization process. Without significantly increasing parameters or FLOPs, this strategy greatly improves detection robustness for small targets, occluded or blurred objects, and dense multi-object scenarios.

AOWIoU applies dynamic sample-level gains to positive samples in the loss layer, thereby allocating more gradient budget to “teachable” medium-quality samples and explicitly boosting the learning weights of occluded and blurred samples. Let the IoU between each positive sample’s prediction bounding box and its matched ground truth be denoted as $q={IoU}_{1}\in [0,1]$(IoU for the predicted bounding box and the matched ground-truth box in positive samples). The maximum IoU between this sample and its “second-best ground truth” within the same image is denoted as $\hat{q}={IoU}_{2}$(maximum Intersection over Union between the sample and the sub-optimal ground-truth box in the same image). Based on this, occlusion and confusion metrics are defined as IoU_AO:


$$IoU\_AO=\max\;(0,\;q-\hat{q}),$$ (2)

$$1_{fuzzy}=1\{\hat{q}>\tau_{fz}\}.$$ (3)

When $\hat{q}$ is large, IoU_AO decreases, indicating that predictions are more easily confused with other objects, suggesting that samples are occluded or have blurred boundaries. To avoid the influence of gradient dominance from high-quality samples in the later stages of training and the “harmful gradients” from low-quality samples, we first introduce a symmetric, decaying medium-quality reinforcement weight, as shown in equation 4.


$$w_{mid}(q)=(q(1-q){)}^{\beta },\;\beta \geq 1,$$ (4)

It peaks at q≈0.4~0.7, naturally emphasizing the medium-quality range. Subsequently, the occlusion and blur perception components are constructed using IoU_AO, adjusting blurred sample weights upward while weighting clear samples based on IoU-perceived positioning:


$$w_{ao}(q,\hat{q})=\left\{ \begin{array}{cc} (2-IoU\_AO{)}^{\gamma_{c}}, & 1_{fuzzy}=1,\\ \delta +q, & 1_{fuzzy}=0, \end{array}\right.$$ (5)

$$\gamma_{c}>0,\;\delta \in \{0,1\}.$$ (6)

Given that extremely low-quality samples are often accompanied by strong noise or poor localization, flexible gating is further introduced to suppress their gradients:


$$w_{low}(q)=\sigma \;\left(\frac{q-\tau_{low}}{s}\right),$$ (7)

Here, $\tau_{low}$ and *s* control the gate start point and slope, respectively.

Combine the three metrics to get a unified sample gain, then perform intra-batch normalization and clip the values at upper and lower bounds to stabilize the loss scale.


$$\tilde{w}=w_{mid}\cdot w_{ao}\cdot w_{low},$$ (8)

$$w=clip\;\left(\frac{\tilde{w}}{mean(\tilde{w})+\varepsilon },\;c_{\min},\;c_{\max}\right).$$ (9)

On the regression side, AOWIoU maintains WIoUv3’s non-monotonic focus and strong geometric constraints, but multiplies each sample’s gradient by *w* to give a larger gradient budget to “teachable regions” and occluded samples.


$${ℒ}_{reg}^{AOWIoU}=mean\;(w\cdot {ℒ}_{reg}^{WIoUv3}(b,\;b^{gt})).$$ (10)

The classification module uses IoU-aware quality learning, employing *q* as soft labels while sharing the same weight vector *w* to reduce confidence bias caused by occlusions.


$${ℒ}_{cls}^{IoU\text{-}aware}=mean\;\left(w\cdot [-(1-|p-q|{)}^{\gamma_{p}}\log(1-|p-q|)]\right).$$ (11)

The ultimate joint objective is:


$$ℒ=\lambda_{reg}{ℒ}_{reg}^{AOWIoU}+\lambda_{cls}{ℒ}_{cls}^{IoU\text{-}aware},$$ (12)

The variables and hyperparameters used in the AOWIoU formula are detailed in Table 1:

**Table 1:** Definitions of symbols used in the loss functions.

Symbol definitions	
$1_{fuzzy}$	A binary indicator function (0 or 1) that flags a sample as ambiguous if $\hat{q}>\tau_{fz}$, where $\tau_{fz}$ is a threshold.
$w_{mid}$	A symmetric weight focusing on medium-quality samples, peaking at *q* ≈ 0.5. Its shape is controlled by the hyperparameter β (β≥1).
$w_{ao}$	The occlusion-aware weight component. It amplifies the weight for fuzzy samples (1_fuzzy_ = 1) using a factor based on IoU_AO and exponent $\gamma_{c}$, while for clear samples (1_fuzzy_ = 0), it scales linearly with *q* plus a bias δ.
$w_{low}$	A gating function based on the Sigmoid function σ(·) that suppresses the gradients of very low-quality samples (with *q* below the threshold $\tau_{low}$). The parameter *s* controls the slope of the suppression.
$\hat{w},w$	The final sample-wise adaptive weight. $\hat{w}$ is the product of *w*_mid_, *w*_ao_, and *w*_low_. *w* is obtained by normalizing $\hat{w}$ within a batch and clipping it to the range $\left[c_{min},c_{max}\right]$ to stabilize training.
${ℒ}_{reg}^{WIoUV3}$	The regression loss value computed by the WIoUv3 function.
${ℒ}_{reg}^{AOWIoU}$	The final AOWIoU regression loss, which is the mean of the WIoUv3 loss modulated by the weight *w*.
${ℒ}_{cls}^{IoU\text{-}aware}$	The classification loss that uses *q* as a soft target and shares the same weighting vector *w* to align the optimization focus between the regression and classification branches. *p* is the predicted class probability.

Where $\lambda_{reg},\lambda_{cls}$ denote branch weights. In practice, $\hat{q}={IoU}_{2}$ can be implemented by grouping images into positive samples, computing pairwise IoU with all ground truth boxes for that image, and taking the maximum “non-matched ground truth” value for each positive sample. This overhead is negligible under mainstream dense detection settings and can be further reduced using local candidate or sparse nearest neighbor approximations. AOWIoU is compatible with CDA, with all modifications occurring only during training, resulting in zero intrusion on inference and suitability for edge deployment. Empirically, $\beta =2.0,\;\gamma_{c}=1.0,\;\tau_{fz}=0.01,\;\delta =1.0,\;\tau_{low}=0.20,\;s=0.08,\;\gamma_{p}=2.0,\;c_{\min}=0.25,\;c_{\max}=4.0$ yield stable performance on the same dataset; For data with more severe occlusions, moderately increase $\gamma_{c}$ or $\tau_{fz}$. If instability occurs early in training, employ an ensemble scheduling approach for β and $\gamma_{c}$, gradually increasing them from small to large values

### SGP

To clarify how SGP relates to existing spatial–channel rearrangement techniques, we first revisit PixelShuffle and its inverse operation, Space-to-Depth (SPD). PixelShuffle—originally introduced as a sub-pixel convolution layer for efficient super-resolution—rearranges feature maps by moving information from the channel dimension into a finer spatial grid, enabling learnable upsampling at low computational cost [26]. SPD performs the opposite permutation: it partitions an input feature map into non-overlapping spatial blocks and stacks them along the channel dimension, thereby reducing spatial resolution while (in principle) preserving all information through a deterministic re-indexing rather than discarding samples. Recently, this idea has been adopted in SPD-Conv as a replacement for strided convolutions and pooling in low-resolution and small-object scenarios, because it mitigates the loss of fine-grained details caused by lossy stride-2 downsampling. Building on this connection, SGP uses SPD as an information-preserving downsampling step and further introduces lightweight grouped convolution to perform learnable, low-cost channel fusion after the spatial-to-channel rearrangement—distinguishing it from using SPD alone and from PixelShuffle-style operators that primarily target upsampling.

**Fig 4:**
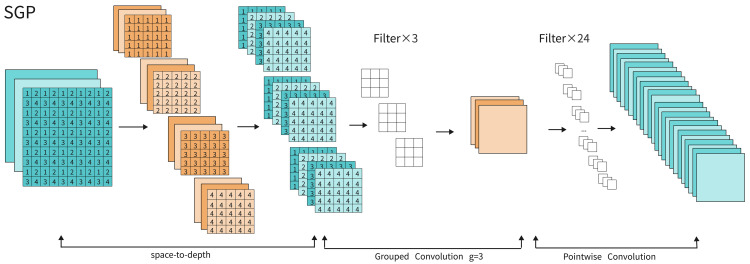
Diagram of SGP.

The SGP module uses a design approach that converts spatial information into depth information. Its core involves mapping the spatial dimensions of input features to depth dimensions, thus removing the need for pooling layers and strided convolutions found in traditional methods. This approach increases the depth of feature maps while retaining as much original feature information as possible, effectively reducing the loss of fine details and improving representational power in low-resolution images or small object detection tasks. The module also lowers computational complexity while preserving model accuracy. The basic implementation of SGP is shown in Fig 4.

**Fig 5:**
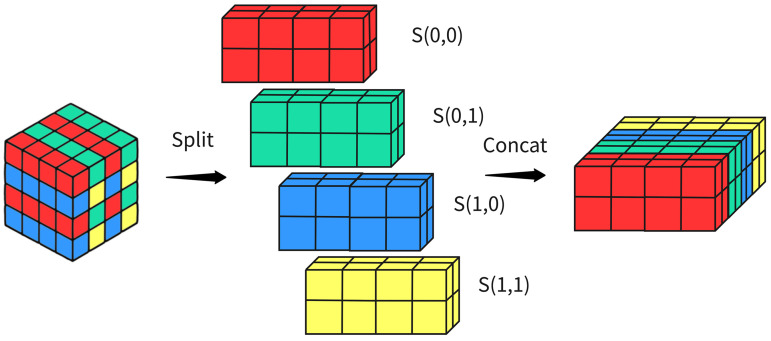
Diagram of space-to-depth. If its input feature map shape is $S,S,C_{1}$, with a scaling factor of scale. It generates a new feature map with shape $\left(S/scale,S/scale,{scale}^{2}\times C_{1}\right)$ (where scale is the scaling factor).

As shown in Fig 5, we define the input feature map X as W×H×C, set the scaling factor to scale = 2, and divide it into four sub-feature maps: S(0,0), S(1,0), S(0,1), S(1,1). Each sub-feature map has dimensions (W/2, H/2, C), corresponding to a 2x spatial downsampling. Subsequently, each sub-feature map is concatenated along the channel dimension to form a new feature map *X*_*ext*1_ with dimensions (W/2, H/2, 4×C). Since the depth-spatial transformation unfolds local spatial blocks before concatenating them along the channel dimension, the sub-feature maps (mean, variance, response scale) often differ significantly. Directly feeding them into convolutions for channel mixing forces the network to learn scale adaptation, increasing training complexity. The increased number of channels after concatenation, coupled with varying data support (smaller spatial dimensions) per channel, can cause abnormal activation value distributions in certain channels. This affects the stability of subsequent activation function computations and optimization, increasing computational complexity. Therefore, we perform grouped convolutions by dividing the input channels into G subsets for independent convolution operations, thereby reducing the number of parameters and computational load.

The core idea of Adaptive Grouped Convolution is to adaptively select an appropriate grouping number G based on the divisibility of the input channel count, and then perform convolution operations independently on each group of channels. Specifically, we designed a “maximum divisibility priority” grouping strategy: First, set an upper limit *G*_max_ for grouping (set to 16 in experiments). Then, while ensuring *G*_max_ is not exceeded, search for the grouping size from largest to smallest. Select the largest value that divides C’ evenly as the final G. This grouping strategy ensures that while reducing parameters and FLOPs, the grouped convolution fully leverages the distribution characteristics among channels. It avoids redundant or ineffective groupings caused by non-divisibility, enabling the convolution operation to maintain high computational efficiency while preventing feature isolation due to excessive segmentation. Mathematically, this can be expressed as:


$$G=\max\{\;g|g\leq G_{\max},\;C^{\prime }\mathrm{mod}g=0\;\}.$$ (13)

**Fig 6:**
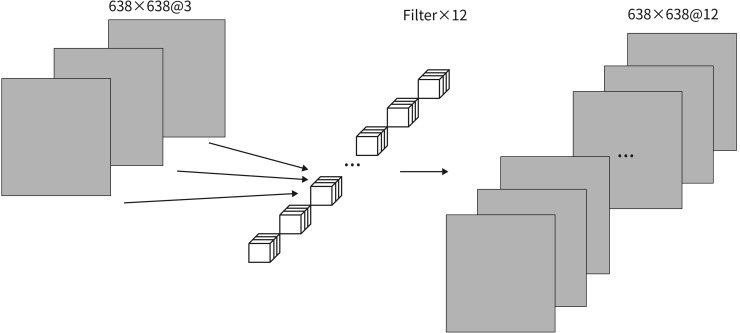
Diagram of Pointwise Convolution.

Although grouped convolutions are highly efficient, their inherent limitations necessitate performing pointwise convolutions in the final step, as shown in Fig 6. This bridges information exchange between channels, enabling cross-channel integration of global and local features. By appropriately setting the number of output channels, pointwise convolutions can scale feature dimensions to the network’s required size. This approach restores expressive power while enhancing feature map adaptability for downstream detection heads.

### LCFNet

In lightweight and edge computing scenarios, standard convolutions combine spatial modeling with cross-channel mixing. The computational complexity per output position is approximately given by $k^{2}C_{\text{in}}C_{\text{out}}$, or about $HWk^{2}C_{\text{in}}C_{\text{out}}$ overall. This makes it difficult to balance accuracy and real-time processing under limited computational resources. To address this, the paper uses a DepthSepConv module as the backbone and introduces an SGP (Spatial Grouped-Pointwise) module in the downsampling path. After global average pooling (GAP), the network’s tail connects to a 1 × 1 pointwise convolution (with 1280 output channels) to expand the channel dimension and perform a linear combination, similar to a fully connected layer. The backbone network’s layer configuration and parameter scale are detailed in Table 2.

**Table 2:** The detailed architecture of LCFNet.

LCFNet Architecture Details					
Operator	Kernel Size	Stride	Input Size	Output Size	SE
Conv	3 × 3	2	640 × 640 × 3	320 × 320 × 8	✗
DepthSepConv	5 × 5	1	320 × 320 × 8	320 × 320 × 8	✗
SGP	3 × 3	1	320 × 320 × 8	160 × 160 × 16	✗
SGP	3 × 3	1	160 × 160 × 16	80 × 80 × 32	✗
SGP	3 × 3	1	80 × 80 × 32	40 × 40 × 64	✗
3×DepthSepConv	5 × 5	1	40 × 40 × 64	40 × 40 × 64	✗
DepthSepConv	5 × 5	2	40 × 40 × 64	20 × 20 × 128	✓
DepthSepConv	5 × 5	1	20 × 20 × 128	20 × 20 × 128	✓
GAP (Global Avg Pooling)	7 × 7	1	20 × 20 × 128	20 × 20 × 128	✗
Conv2D + NBN	1 × 1	1	1 × 1 × 128	1 × 1 × 1280	✗

Table notes: SGP denotes the proposed downsampling module; SE indicates whether the Squeeze-and-Excitation block is enabled (✓) or not (✗).

DepthSepConv employs depth-wise convolutions (DW) to focus on local spatial patterns within each channel, followed by point-wise convolutions (PW / 1 × 1) to perform cross-channel linear combinations and channel-to-channel mapping. Furthermore, activation functions remain a critical bottleneck on resource-constrained devices: While ReLU6 [27] offers low computational cost, its zero derivative in the saturation region for *x* > 6 can cause vanishing gradients; Swish [28] (xσ(x)) provides curve smoothing but incorporates exponential operations, resulting in high inference costs. Balancing training stability and inference efficiency, we uniformly adopts the Hardswish activation—a piecewise linear approximation of Swish—after DW and PW in this paper. Its formula is:


$$Hardswish(x)\;=\;\frac{x\cdot ReLU6(x+3)}{6},$$ (14)

ReLU6(x)=min{max(0,x),6}. (15)

Hardswish avoids exponential operators while maintaining smoothness and boundedness, making it more suitable for lightweight networks and edge devices.

Given an input tensor $X\in {\mathbb{R}}^{H\times W\times C_{in}}$, DepthSepConv first performs spatial convolution independently on each input channel to obtain an intermediate tensor *Z*. Let the convolution kernel size be *k* × *k* with stride *s* (set to *s* = 1 in this context), yielding an output spatial dimension of $H^{\prime }\times W^{\prime }$. The DW formula is:


$$Z_{c}(i,j)\;=\;\sum_{u,v}K_{c}^{\left(d\right)}(u,v)\;X_{c}(i+u,\;j+v),$$ (16)

$$c=1,\ldots ,C_{in},$$ (17)

$$(i,j)\in [1,H^{\prime }]\times [1,W^{\prime }].$$ (18)

Subsequently, PW performs a 1 × 1 convolution to complete the linear combination and channel dimension mapping in the channel dimension, yielding the output $Y\in {\mathbb{R}}^{H^{\prime }\times W^{\prime }\times C_{out}}$:


$$Y_{c^{\prime }}(i,j)\;=\;\sum_{c=1}^{C_{in}}K^{\left(p\right)}(c,c^{\prime })\;Z_{c}(i,j),$$ (19)

$$c^{\prime }=1,\ldots ,C_{out},$$ (20)

$$\;\;(i,j)\in [1,H^{\prime }]\times [1,W^{\prime }].$$ (21)

Compared to the computational complexity of standard convolution, $H^{\prime }W^{\prime }k^{2}C_{in}C_{out}$, the complexity of DepthSepConv is the sum of the DW and PW components:


$$\underset{\text{DW}}{\underbrace{H^{\prime }W^{\prime }\;C_{in}k^{2}}}\;+\;\underset{\text{PW}}{\underbrace{H^{\prime }W^{\prime }\;C_{in}C_{out}}},$$ (22)

Accordingly, the number of parameters is reduced from $k^{2}C_{in}C_{out}$ to $C_{in}k^{2}+C_{in}C_{out}$.

To enhance channel discriminative power, lightweight channel attention (SE) channels [29] can be inserted between DW and PW as needed for re-calibration (commonly using two nonlinear layers: ReLU and H-Sigmoid, where Hsigmoid(x)=ReLU6(x+3)/6). Regarding convolution kernel sizes, to avoid memory access and parallelization overhead from mixing multiple kernels within the same layer, we maintains a single kernel size per module. When stronger local context is required, the 3 × 3 DW kernel can be moderately replaced with a 5 × 5 kernel. Leveraging the decomposed structure allows for a larger effective receptive field with minimal additional computational complexity.

## Experiments

### Datasets and experimental environment

In this paper, the Smoking Detection Dataset is used to evaluate the performance of the developed AOW-YOLO. The Smoking Detection Dataset [30] is a public dataset consisting of images capturing smoking behaviors across various scenarios. It contains a total of 18,160 images, each measuring 640 × 640 pixels. The object types in the Smoking Detection Dataset include cigarettes. Due to its original imbalanced sample distribution, we used a random assignment strategy to split the dataset into training, validation, and test sets at an 8:1:1 ratio, resulting in 14,528, 1,816, and 1,816 images, respectively.

The training environment setup is detailed in Table 3. The computer features an Intel(R) Xeon(R) Platinum 8352V processor (2.10 GHz) and an NVIDIA GeForce RTX 4090 24G GPU. It runs Windows 11 Professional. The development environment was established using Anaconda with Python 3.10.16 and the PyTorch 2.6.0 deep learning framework. Moreover, the experiments utilized the CUDA 12.4 platform and cuDNN 9.1.0 neural network library for acceleration.

**Table 3:** Experimental Hardware and Software Configuration.

Hardware & Software Settings	
Name	Configuration
CPU	Intel(R) Xeon(R) Platinum 8352V CPU @ 2.10GHz
GPU	NVIDIA GeForce RTX 4090 24G
Operating System	Windows 11 Professional Edition
Deep Learning Framework	PyTorch 2.6.0
Development Environment	Python 3.10.16, CUDA 12.4
GPU-accelerated Library	cuDNN 9.1.0

Table notes: All experiments were conducted under the same hardware and software configuration.

We uses the Adam optimizer with a decay coefficient of 0.0005 for model training. The batch size is 64, and the initial learning rate is 0.001.

### Evaluation indicators

In this paper, we comprehensively evaluate object detection models using metrics such as mAP50, mAP50:95, FLOPs, Params, and FPS. Among these, mAP is calculated based on the area under the precision-recall (PR) curve (AP), averaging the AP values across all categories. Since this paper includes only a single category, mAP is equivalent to the AP value for that category. mAP50 denotes the AP calculated at a fixed IoU threshold of 0.50. mAP50:95 involves computing AP at 10 IoU thresholds from 0.50 to 0.95 in steps of 0.05 (0.50, 0.55, ..., 0.95) and then averaging these values. This metric is more sensitive to localization quality and typically produces lower values than mAP50, better reflecting the robustness of detection boxes under varying overlap requirements. Regarding computational complexity, FLOPs and Params measure model scale and theoretical computational load: FLOPs represent the number of floating-point operations required for a single inference (usually approximated by multiply-accumulate operations), while Params indicate the number of trainable parameters. FPS provides an intuitive measure of inference speed, while FLOPs serve as an indirect indicator of computational load. Positive and negative samples are classified based on IoU scores: IoU ≥ 0.50 indicates a positive sample, and IoU < 0.50 indicates a negative sample. To improve statistical robustness, all metrics in this study are derived from 10 independent repetitions, with the highest and lowest values discarded and the remaining results averaged. Considering the computational constraints of edge devices at construction sites, the proposed AOW-YOLO is deployed and tested on an industrial control computer environment. All test data are collected from this actual deployment (hardware configuration details are shown in Table 4).

**Table 4:** Test Condition.

Testing Environment Configuration	
Name	Configuration
CPU	Intel(R) Core(TM) i3-10110U CPU @ 2.10GHz
Operating System	Windows 10 Professional Edition
Deep Learning Framework	PyTorch 2.6.0
Development Environment	Python 3.10.16
INT8 Quantization	NO
Batch Size	1

Table notes: All models were evaluated under identical test conditions on the same CPU platform.

### Comparison of loss functions

To validate the effectiveness of the AOWIoU loss function introduced in the detection head, we conducted a comparative analysis of existing mainstream bounding box regression loss functions, including GIoU, DIoU, CIoU, SIoU, EIoU, WIoUv1, WIoUv2, and WIoUv3. Since loss functions only affect gradient allocation and backpropagation during training, they have no significant impact on model parameters (Params), computational complexity (FLOPs), or inference speed (FPS). Therefore, this experiment mainly analyzes detection accuracy and convergence speed.

As shown in Table 5, among the traditional IoU series loss functions, CIoU demonstrates superior performance with mAP50 = 66.8% and mAP50:95 = 33.5%, exhibiting stable localization abilities. However, it converges relatively slowly and is prone to gradient saturation in the mid-to-late training phases. Within the WIoU family, WIoUv3 shows better accuracy through its dynamic sample weighting mechanism, achieving mAP50 = 66.5% and mAP50:95 = 32.9%. It also demonstrates a faster reduction in box_loss during the early training stages, indicating advantages over traditional IoU variants in gradient distribution and convergence stability.

**Table 5:** Comparison of different loss functions.

Loss Function Comparison		
Method	mAP50 / %	mAP50:95 / %
CIoU	66.8	33.5
DIoU	64.2	31.8
GIoU	65.8	32.8
SIoU	66.0	32.9
EIoU	66.1	33.0
WIoUv1	65.8	32.4
WIoUv2	64.8	31.6
WIoUv3	66.5	32.9
AOWIoU	**68.8**	**34.4**

Table notes: The best results are highlighted in bold.

**Fig 7:**
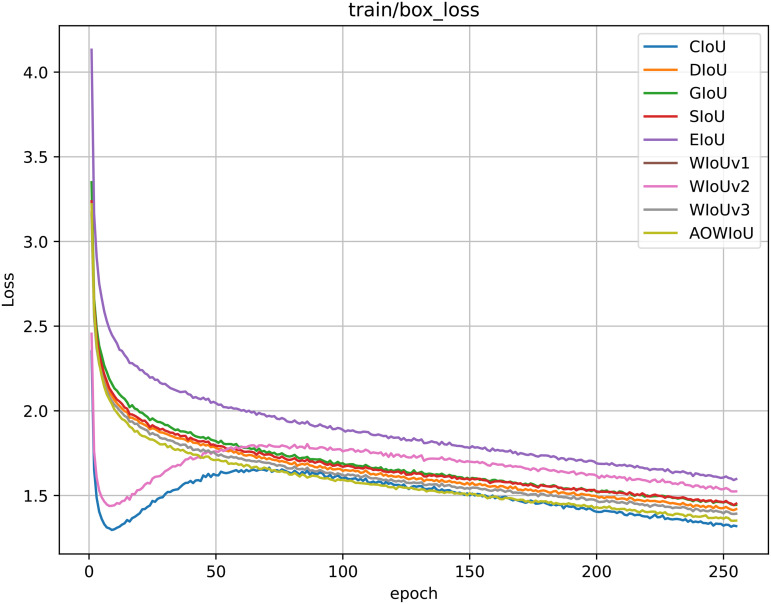
Comparison of Convergence Rates Across Loss Functions.

Building on this foundation, the proposed AOWIoU achieves optimal performance across three metrics: mAP50 reaches 68.8%, and mAP50:95 reaches 34.4%, representing improvements of 3.6% and 1.9% over WIoUv3, respectively. Compared to traditional loss functions like CIoU and DIoU, AOWIoU not only significantly enhances detection accuracy but also converges more quickly during training. As shown in the loss curve in Fig 7, AOWIoU’s box_loss decreases at a notably faster rate than other methods, indicating better stability and robustness in scenarios with complex occlusions and small targets.

This advantage mainly comes from AOWIoU’s use of occlusion-aware components and strategies to improve medium-quality samples, based on the dynamic focus mechanism of WIoUv3. These innovations reduce interference from very high- or low-quality samples during gradient updates while increasing the learning weight of blurred and partially occluded samples. This allows for adaptive reinforcement learning with “trainable samples,” greatly improving the detection head’s ability to localize and classify.

The comprehensive results show that AOWIoU greatly improves the model’s ability to distinguish complex samples and ensures stable optimization while keeping computational costs low. Its dynamic fuzzy sample weighting and occlusion-aware mechanism effectively address the performance issues of traditional IoU variants in situations with occlusion, blurring, and small targets. This provides a new approach to making lightweight detection models more robust in challenging environments.

### Ablation study

This study aims to perform ablation experiments on the lightweight model YOLO11n using multiple optimization strategies to examine the combined effects of LCNet, SGP, and AOWIoU on model performance, efficiency, and complexity. Eight control groups were designed (as shown in the Table 6), comprehensively assessing performance across four dimensions: model accuracy (mAP50, mAP50:95), inference speed (FPS), parameters (Params), and computational load (FLOPs). Overall, the experimental data clearly demonstrate the trade-offs introduced by different technical modules: LCNet functions as the core of model lightweighting, significantly reducing the parameters and computational load but with some accuracy loss; SGP acts as a powerful speedup accelerator, greatly increasing FPS while also positively influencing accuracy; AOWIoU serves as an effective module for improving detection accuracy, robustly enhancing performance with almost no additional computational cost.

**Table 6:** Ablation study of AOW-YOLO.

Ablation Settings					Performance Metrics				
	YOLO11n	LCNet	SGP	AOWIoU	mAP50/%	mAP50:95/%	FPS	Params/M	FLOPs/G
1	✓	×	×	×	72.2	37.4	6.56	2.58	6.3
2	✓	✓	×	×	66.3	32.2	9.37	1.52	3.9
3	✓	×	✓	×	73.0	38.4	5.04	2.43	7.1
4	✓	×	×	✓	73.4	37.1	6.58	2.58	6.3
5	✓	✓	×	✓	67.2	33.2	9.24	1.52	4.1
6	✓	×	✓	✓	72.3	38.4	4.84	2.44	7.1
7	✓	✓	✓	×	68.2	33.8	8.50	1.52	3.9
8	✓	✓	✓	✓	**68.8**	**34.4**	**8.63**	**1.53**	**4.1**

Table notes: ✓ indicates enabled, and × indicates disabled. The best results are highlighted in bold. LCNet: a lightweight backbone network (its original downsampling strategy is used by default in the ablation studies); SGP: a module that replaces the backbone’s downsampling convolutions; AOWIoU: an adaptive regression loss function; LCFNet: a backbone formed by integrating LCNet with SGP. Evaluation metrics encompass mAP50, mAP50:95, FPS, Params, and GFLOPs.

A thorough analysis of each module’s mechanism clearly shows the performance changes and trade-offs it causes. First, the addition of LCNet (comparing Experiments 1 and 2, 3 and 7, 4 and 5, 6 and 8) is a key factor in making the model lighter. For example, after adding LCNet (Experiment 1 vs. Experiment 2), the number of parameters dropped from 2.58 million to 1.52 million (about 41% reduction), FLOPs decreased from 6.3 G to 3.9 G (about 38% decrease), and inference speed (FPS) improved from 6.56 to 9.37 (about 43% increase). However, this significant efficiency gain comes with a decrease in accuracy, as mAP50 dropped from 72.2% to 66.3%, showing that LCNet reduces some feature representation ability while cutting computational costs.

Secondly, the role of the SGP module mainly involves balancing inference speed and feature accuracy. When SGP was used alone (Experiment 1 vs. Experiment 3), FPS dropped from 6.56 to 5.04, showing that SGP adds computational overhead without making the model lighter. However, when combined with LCNet (Experiment 2 vs. Experiment 7), FPS decreased from 9.37 to 8.50, indicating that SGP can help improve computational efficiency in lightweight models, boosting real-time performance on edge devices. At the same time, SGP usually causes small changes in accuracy. For example, mAP50 went from 72.2% to 73.0% between Experiments 1 and 3, and from 66.3% to 68.2% between Experiments 2 and 7. This helps partially offset the accuracy loss from lightweighting.

Finally, the AOWIoU loss function (as shown in Experiments 1 vs. 4, 2 vs. 5, 3 vs. 6, and 7 vs. 8) acts as an advanced regression optimization method, delivering notable accuracy gains with minimal computational cost. After applying AOWIoU across all baselines (e.g., Experiment 1 vs. 4, Experiment 7 vs. 8), mAP50 increased by 0.7%–1.6%, and mAP50:95 rose by 0.4%–0.9%, while parameters and FLOPs stayed mostly unchanged. This clearly proves its plug-and-play efficiency.

From the viewpoint of different activation configurations, Table 6 also reveals how single-module and combined-module settings shift the balance among accuracy, efficiency, and complexity. When only one module is enabled (Experiments 2–4), each technique mainly optimizes a single aspect: LCNet (Experiment 2) aggressively compresses parameters and FLOPs and pushes FPS to 9.37, but sacrifices 5.9% mAP50 compared with the baseline; SGP alone (Experiment 3) slightly reduces parameters yet increases FLOPs and yields a moderate FPS drop, while bringing a modest gain of 0.8%/1.0% in mAP50/mAP50:95; AOWIoU alone (Experiment 4) keeps Params and FLOPs almost unchanged and mainly improves accuracy. In contrast, two-module combinations (Experiments 5–7) show that LCNet and SGP jointly maintain a highly lightweight backbone (1.52 M, 3.9 GFLOPs) with better accuracy than LCNet alone, while adding AOWIoU on top of LCNet or LCNet + SGP (Experiments 5 and 8) further recovers part of the accuracy loss at nearly no additional complexity. These trends indicate that single-module settings tend to move the model along one axis of the accuracy–efficiency spectrum, whereas combined configurations, especially those including AOWIoU, achieve a more favorable Pareto trade-off under strict resource constraints.

A comprehensive comparison of all experimental results shows that Experiment 8 (LCNet + SGP + AOWIoU) offers the best balance between speed and accuracy. This setup maintains very low computational overhead (1.53 million parameters, 4.1 GFLOPS) while achieving an inference speed of 8.63 FPS and detection accuracy of 68.8% / 34.4%, demonstrating excellent lightweight performance and suitability for edge deployment. It is the ideal configuration for real-time detection tasks like construction sites.

#### Comparison of different detection models.

To ensure comparability, this section selects two representative baselines for comparison under unified datasets, training strategies, and inference settings: general lightweight detectors (YOLO11n, YOLOv8n) and typical lightweight backbones (PP-LCNet, ShuffleNetv2, GhostNet, FasterNet). Evaluation metrics include mAP50, mAP50:95, FPS, Params, and GFLOPs. The results are shown in Table 7.

**Table 7:** Comparison of different models.

Model	Accuracy		Efficiency		
	mAP50/%	mAP50:95/%	FPS(CPU-only)	Params/M	FLOPs/G
AOW-YOLO	**68.8**	**34.4**	**8.63**	**1.53**	**4.1**
YOLO11n	72.2	37.4	6.56	2.58	6.3
YOLOv8n	71.4	37.2	5.22	2.69	8.2
YOLO12n	72.8	36.8	6.32	2.56	6.3
PP-LCNet	66.3	32.2	9.37	1.52	3.9
ShuffleNetv2	59.4	28.6	8.31	1.82	5.4
GhostNet	59.5	28.7	5.31	2.92	5.3

Table notes: All models are evaluated under the same test conditions (Table 4).

With only 1.53 million parameters and a computational budget of 4.1 GFLOPs, AOW-YOLO achieves 68.8% mAP50, 34.4% mAP50:95, and an inference speed of 8.63 FPS. Compared to YOLO11n, mAP50 decreased by 3.4 percentage points and mAP50:95 by 3.0 percentage points, but the frame rate increased by 31.5% (8.63 vs 6.56 FPS). At the same time, the number of parameters and FLOPs were reduced by 40.7% and 34.9%, respectively. Compared to YOLOv8n, AOW-YOLO shows a slightly lower mAP50 by 2.6% and mAP50:95 by 2.8%, yet it achieves a 65.3% higher frame rate (8.63 vs 5.22 FPS), while lowering parameters and FLOPs by 43.1% and 50.0%, respectively. This demonstrates superior computational efficiency and real-time performance in scenarios with low computational power.

In a comparative analysis with lightweight backbone networks, AOW-YOLO outperforms PP-LCNet by achieving a 2.5% higher mAP50 and a 2.2% increase in mAP50:95. This enhanced detection accuracy and stability come with only a slight increase in FLOPs (4.1G compared to 3.9G). When compared to ShuffleNetv2, AOW-YOLO shows a 9.4 percentage point boost in mAP50 and a 5.8 point increase in mAP50:95 at similar computational levels. Although the FPS increases marginally from 8.31 to 8.63 (about 3.9%), it indicates a better balance between accuracy and speed. Against GhostNet, AOW-YOLO delivers a 9.3% improvement in mAP50 and a 5.7% increase in mAP50:95, with more efficient inference due to fewer parameters (1.53M vs. 2.92M) and lower computational cost (4.1G vs. 5.3G).

From an overall efficiency perspective, AOW-YOLO strikes an optimal balance between accuracy and real-time performance with limited computational resources. While keeping a lightweight architecture, the model improves both detection performance and energy efficiency through information-preserving subsampling (SGP) and adaptive regression loss (AOWIoU). This confirms its effectiveness and engineering value for real-time smoking detection in complex construction environments.

**Fig 8:**
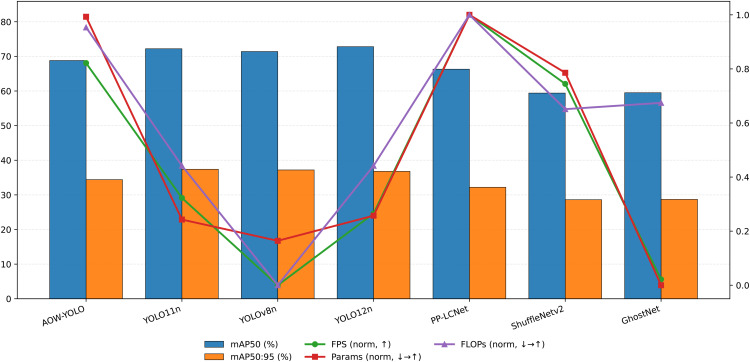
Model Inference Results.

**Fig 9:**
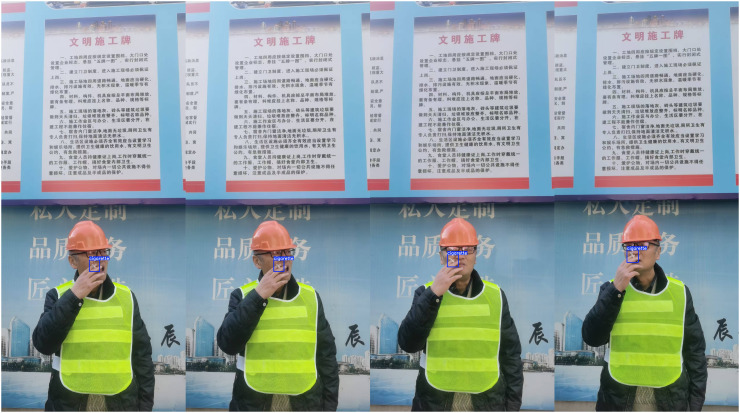
Detection examples of AOW-YOLO. [The individual in this manuscript has given written informed consent (as outlined in PLOS consent form) to publish this image].

To provide a more intuitive comparison of different model performances, the experiment evaluated seven models including AOW-YOLO and YOLO11n based on three key metrics: mAP50, mAP50:95, and FPS. The results are shown in Fig 8. Finally, we present the test results. The industrial control computer configuration is detailed in Table 4. The results of the deployed model are shown in Fig 9.

## Conclusions

This paper introduces the lightweight and efficient AOW-YOLO for accurate identification and real-time early warning of smoking behavior in complex construction environments. The model uses the lightweight backbone LCFNet, employs SGP subsampling to maintain fine-grained details, and applies AOWIoU loss combined with Consistency Dual-Label Assignment (CDA) in the decoupled detection head to more stably optimize medium-quality and challenging samples. Experiments show that, compared with YOLO11n and YOLOv8n, AOW-YOLO achieves substantially higher CPU inference speed and lower computational cost with an acceptable accuracy trade-off, making it more suitable for resource-constrained edge deployment. It demonstrates increased robustness and suitability for edge deployment in typical construction site scenarios featuring small targets, severe occlusions, and high target density, confirming the combined effectiveness and practical value of its enhancements.

Future work will focus on more challenging areas: First, improving cross-site, cross-time, and cross-climate distribution generalization and out-of-domain robustness, combined with data-efficient learning approaches to reduce annotation costs and mitigate data drift. Second, enhancing efficiency and model reliability under strict computational and energy constraints (e.g., energy-efficient adaptive inference and uncertainty estimation) to ensure stable and trustworthy early warnings. Third, developing temporal and multimodal extensions that leverage temporal correlations and heterogeneous sensor data to increase robustness against occlusions, jitter, and extreme lighting conditions while maintaining a lightweight architecture. These advancements will help accelerate the large-scale deployment of AOW-YOLO in smart construction site safety inspections, urban security, and industrial site violation monitoring.

## Supporting information

S1 File Illustration of the loss function. Visualization of the loss function. Generate the raw data for the line chart in Fig 7.
(XLSX)

S1 Text URL: https://universe.roboflow.com/kyunghee-university-ada5d/smoking-detection-3gefl.
(TXT)
